# CINeMA: Software for semiautomated assessment of the confidence in the results of network meta‐analysis

**DOI:** 10.1002/cl2.1080

**Published:** 2020-03-11

**Authors:** Theodoros Papakonstantinou, Adriani Nikolakopoulou, Julian P. T. Higgins, Matthias Egger, Georgia Salanti

**Affiliations:** ^1^ Institute of Social and Preventive Medicine University of Bern Bern Switzerland; ^2^ Population Health Sciences, Bristol Medical School University of Bristol Bristol UK

## Abstract

Network meta‐analysis (NMA) compares several interventions that are linked in a network of comparative studies and estimates the relative treatment effects between all treatments, using both direct and indirect evidence. NMA is increasingly used for decision making in health care, however, a user‐friendly system to evaluate the confidence that can be placed in the results of NMA is currently lacking. This paper is a tutorial describing the Confidence In Network Meta‐Analysis (CINeMA) web application, which is based on the framework developed by Salanti et al (2014, *PLOS One*, 9, e99682) and refined by Nikolakopoulou et al (2019, *bioRxiv*). Six domains that affect the level of confidence in the NMA results are considered: (a) within‐study bias, (b) reporting bias, (c) indirectness, (d) imprecision, (e) heterogeneity, and (f) incoherence. CINeMA is freely available and open‐source and no login is required. In the configuration step users upload their data, produce network plots and define the analysis and effect measure. The dataset should include assessments of study‐level risk of bias and judgments on indirectness. CINeMA calls the netmeta routine in R to estimate relative effects and heterogeneity. Users are then guided through a systematic evaluation of the six domains. In this way reviewers assess the level of concerns for each relative treatment effect from NMA as giving rise to “no concerns,” “some concerns,” or “major concerns” in each of the six domains, which are graphically summarized on the report page for all effect estimates. Finally, judgments across the domains are summarized into a single confidence rating (“high,” “moderate,” “low,” or “very low”). In conclusion, the user‐friendly web‐based CINeMA platform provides a transparent framework to evaluate evidence from systematic reviews with multiple interventions.

## 1 INTRODUCTION

Network meta‐analysis (NMA) is increasingly being used to make decisions about optimal interventions in health care (Kanters et al., 2016; Petropoulou et al., 2016). It combines direct evidence from studies that directly compare two or more interventions, and indirect evidence from studies that indirectly inform a comparison through intermediate comparators. To evaluate the confidence in the results of NMA, a framework has been developed (Salanti, Del Giovane, Chaimani, Caldwell, & Higgins, 2014) and recently refined (Nikolakopoulou et al., 2019), which is called CINeMA (Confidence In Network Meta‐Analysis). The CINeMA framework considers six domains that affect the level of confidence in the NMA results: (a) within‐study bias, (b) reporting bias, (c) indirectness, (d) imprecision, (e) heterogeneity, and (f) incoherence. Reviewers assess the level of concerns for each relative treatment effect from NMA as giving rise to “no concerns,” “some concerns,” or “major concerns” in each of the six domains. Then, judgments across the domains are summarized into a single confidence rating (“high,” “moderate,” “low,” or “very low”).

The six domains include considerations pertaining to all stages of the systematic review, including literature search, data extraction, and statistical analysis. Within‐study bias domain refers to limitations in the individual studies that may lead to a biased estimated relative treatment effect. Reporting bias results from the inclusion in the systematic review of a nonrepresentative set of the eligible studies, that may occur for example from an uncomplete literature search. Indirectness refers to the relevance of the included studies to the research question, which includes the definition of the population, interventions, and outcomes of interest. A core assumption in NMA is that of transitivity; that there is an underlying true relative treatment effect which applies to all studies regardless of the treatments being compared. Assessment of transitivity is challenging and is usually done by exploring the distribution of effect modifiers per comparison. CINeMA's approach for indirectness intends to also address the assumption of transitivity by indicating which comparisons may suffer from different definitions of the setting of interest. Assuming that transitivity holds implies that consistency—which refers to the agreement of the estimated treatment effects—also holds. This can be assessed under the incoherence domain in CINeMA. Finally, imprecision and heterogeneity domains refer to the certainty with which each effect is estimated and the variability in the results of studies contributing to each comparison respectively.

The CINeMA framework has been implemented in a user‐friendly web application (see https://cinema.ispm.unibe.ch/; CINeMA, 2017). From a technical point of view, CINeMA is a single page application which communicates to an R back‐end server; in particular, the packages meta and netmeta are used (Rücker, Schwarzer, Krahn, & König, 2016; Schwarzer, 2019). It is developed as a custom functional reactive framework and written in JavaScript and PureScript. CINeMA does not permanently store the data, or any other information related to the uploaded projects; only temporary storage takes place for the sake of the calculations or network efficiency. The source code of CINeMA can be found in (Papakonstantinou).

The methodology described in (Nikolakopoulou et al., 2019) has been implemented in CINeMA using “rules” that can automate derivation of domain‐specific judgments. Three rules can be used to summarize the risk of within‐study bias and of indirectness for each relative effect estimate and produce automated judgments. Two levels of judgment for reporting bias are suggested, based on completeness of the literature search, empirical studies, and statistical analyses. The rules for judging imprecision and heterogeneity are based on whether the confidence interval or prediction interval includes the line of no‐effect and prespecified clinically important treatment effects. The use of rules is optional, and the outputs can be partially or fully overridden. However, the semiautomated process helps researchers to form judgments. Early applications of CINeMA have appeared in the literature (Cipriani et al., 2018; Schwingshackl et al., 2018).

Here we provide a tutorial describing the functionality of CINeMA. We explain how the software works, the data formats and requirements, the default options implemented in the rules, and their rationale. We describe the functionality of CINeMA and illustrate its use with the example of a NMA that compared the incidence of diabetes in patients taking antihypertensive drugs or placebo. The network included 22 randomized trials which evaluated the differences between angiotensin‐converting‐enzyme inhibitors (ACE), angiotensin‐receptor blockers (ARB), calcium‐channel blocker (CCB), Beta Blocker, diuretics, and placebo. The NMA found that the risk of diabetes was lower with ARB, and higher with diuretics than with placebo (Elliott and Meyer, 2007). In this example, data on study‐level indirectness only serve to illustrate how the indirectness domain is assessed and do not reflect the relevance of each study to the research question.

## 2 UPLOADING DATA: *MY PROJECTS*

Under “My Projects,” users upload a .csv file with the study outcome data for their project. The dataset should also include the data on the study‐level risk of bias (RoB) and judgments on indirectness. Study‐level RoB would normally summarize considerations on selection, performance, attrition, detection, and reporting bias (Higgins et al., 2011), while study‐level indirectness refers to deviations between the data and the targeted research question. Study‐level RoB and indirectness can take either {1, 2, 3}, {l, m, h} or {L, M, H} values for low, moderate, and high RoB and indirectness respectively.

The outcome can be binary or continuous and the format of the data can be either long or wide. Currently, other types of outcomes, for example, time to event or rate data, are not supported. In the long format each row represents a study treatment arm. For example, two‐arm studies occupy two rows, three‐arm studies occupy three rows, and so on. In the wide format each row represents a study treatment comparison. This corresponds to $\left(\begin{array}{c} T\\ 2 \end{array}\right)$ rows for a T arm study. For binary outcomes, number of events and sample size per treatment arm and study should be provided. For continuous outcomes, means, standard deviations, and sample size per treatment arm and study are needed. Table 1 illustrates how binary and continuous outcomes can be imported using long or wide formats. For example, binary outcome data on long format should be imported as in Table 1a, providing at least six columns for the study id and the treatment name, the events, the sample size, RoB and indirectness per study arm. Table 1 can be used as a guide on how outcome data of different possible formats can be used as input to CINeMA.

**Table 1:** Examples of four possible data formats that can be used as input to CINeMA

	Long format							Wide format										
Binary	Table 1a							Table 1b										
	id	t	r	n	rob	Indirectness		id	t1	r1	n1	t2	r2	n2	rob	Indirectness		
	1	A	5	12	2	1		1	A	5	12	B	7	15	2	1		
	1	B	7	15	2	1		2	A	6	9	B	7	10	3	2		
	2	A	6	9	3	2		2	A	6	9	C	2	8	3	2		
	2	B	7	10	3	2		2	B	7	10	C	2	8	3	2		

Continuous	Table 1c							Table 1d										
	id	t	y	sd	n	rob	Indirectness	id	t1	y1	sd1	n1	t2	y2	sd2	n2	rob	Indirectness
	1	A	5	5	12	2	1	1	A	5	5	12	B	7	6	15	2	1
	1	B	7	6	15	2	1	2	A	6	7	9	B	7	8	10	3	2
	2	A	6	7	9	3	2	2	A	6	7	9	C	2	9	8	3	2
	2	B	7	8	10	3	2	2	B	7	8	10	C	2	9	8	3	2

*Note*: Data should be imported as a .csv file. The displayed column names are the default expected names; if other names are provided CINeMA will return a query. id specifies the study; t specifies the treatment (numeric or string); r is the number of events; n is the sample size; t1 and t2 specify the treatment codes (numeric or string); r1 and r2 are the number of events in treatments t1 and t2; n1 and n2 are the sample sizes in treatments t1 and t2, respectively; y is the mean; sd is the standard deviation; n is the sample size; y1 and y2 are the means in treatments t1 and t2; sd1 and sd2 are the standard deviations in treatments t1 and t2; rob specifies risk of bias and indirectness specifies level of indirectness; rob and indirectness can take either 1, 2, and 3 or L, M, H values for low, moderate, and high risk of bias or level of indirectness.

It might be that summary data are not available for each intervention group for each study. In this situation data can be imported in “inverse variance” format, where a comparison‐specific estimate of the relative treatment effect (assumed to follow a normal distribution) and its standard error are reported (e.g., log odds ratios, standardized mean differences, etc., see Table 2). When the “inverse variance” format is used, CINeMA will prompt the user to define whether the outcome is binary or continuous. Table 2 can be used as a guide on how outcome data of “inverse variance” format can be used as input to CINeMA.

**Table 2:** Example of “inverse variance” data format in CINeMA

id	t1	t2	effect	se	rob	Indirectness
1	A	B	0.5	0.3	2	1
2	B	D	0.8	0.2	1	2
3	A	6	0.7	0.4	3	3

*Note*: Data should be imported as a .csv file. *id* specifies the study, *t1* and *t2* specify the treatment codes (numeric or string), *effect* is the effect estimate of t1 versus t2 which can be log odds ratio, log risk ratio, log hazard ratio, mean difference or standardized mean difference, *se* is the standard error of the effect estimate, *rob* specifies risk of bias, and *indirectness* specifies level of indirectness; rob and indirectness can take either 1, 2, and 3 or L, M, H values for low, moderate, and high risk of bias or level of indirectness.

Users should choose one of the five data formats in Tables 1 and 2. The names of variables can be as in Tables 1 or 2 (in which case CINeMA will automatically recognize which column refers to which variable) but custom field names are also allowed (e.g., “events” instead of “r” for number of events in Table 1a). If custom field names are used, CINeMA will prompt the user to specify which column represents which field after uploading the dataset. Once the procedure is done (or directly after uploading the data, if variable names are exactly as in Tables 1 or 2), information on the file format (long, wide), outcome type (binary, continuous), number of studies, number of interventions, and number of comparisons with direct evidence appears. Renaming the project's title is also possible under *“Rename*.” Then, users can click on “Proceed” to go to “Configuration.”

### 2.1 Worked example

Uploading the network of antihypertensive drugs, CINeMA recognizes the file format (long) and outcome type (binary) and provides summary of the dataset: it includes 22 studies, 6 interventions, and 14 comparisons with direct data.

## 3 SETTING‐UP THE NMA: *CONFIGURATION*

The “Configuration” tab is activated once the dataset has been uploaded and variable names have been successfully defined. In this tab the user needs to define the NMA analysis and is presented with a network plot. This page also allows users to evaluate only a subset of all possible intervention comparisons.

### 3.1 Network plot

The network plot corresponding to the uploaded dataset is automatically drawn with equally sized nodes and edges. Users can choose to weight nodes and/or edges according to the sample size or the number of studies (under “Node size by” and “Edge width by”). Nodes can either be all blue or colored according to the proportion of studies with low (green), moderate (yellow), and high (red) RoB or indirectness (under “Node color by”). “Edge color by” dropdown menu allows coloring edges according to the most prevalent bias level within each comparison (“Majority RoB”), the average RoB of the included studies (“Average RoB”), or the maximum bias level within each comparison (“Highest RoB”); the respective categories for indirectness are also available (“Majority Indirectness,” “Average Indirectness,” “Highest Indirectness”). Different representations may be chosen according to users' interests: for example, “Highest RoB” or “Highest Indirectness” could be chosen when users are interested in viewing the worst pieces of evidence feeding into each comparison. The network plot image can be exported as a .png file using the “Save Plot” button. The outcome data appear next to the network plot. By clicking on a specific edge or node, the respective outcome data corresponding to that edge or node appear on the data table.

### 3.2 Define your analysis

Here users are asked to choose whether to perform a fixed effect or a random effects NMA (under “Analysis model”) and to define effect measure type (under “Effect measure”). For binary outcomes, the options “Odds Ratio,” “Risk Ratio,” and “Risk Difference” will appear, and for continuous outcomes the options “Mean Difference” and “Standardized Mean Difference.”

### 3.3 Select intervention comparisons for evaluation

An NMA that compares several interventions produces estimates for all possible relative effects. However, it can be the case that not all of them are of interest (e.g., comparisons between placebo and older drugs that are no longer used). CINeMA offers the option to select which intervention comparisons are to be evaluated. Users should first select the interventions of interest and then specify whether they want to evaluate all the comparisons that contain these interventions (“Containing any of the above interventions”) or only the comparisons that are formed between the selected interventions (“Between the above interventions”). For example, in a network with four interventions A, B, C, and D selecting A and B with the “Containing any of the above interventions” option will result in evaluation of comparisons AB, AC, AD, BC, and BD (all possible comparisons except CD). Selecting A and B with “Between the above interventions” option will result in evaluating a single comparison (AB). A list of the comparisons to be evaluated then appears. Note that the analysis is performed using all studies irrespective of whether a subset or all comparisons are evaluated.

After defining the comparisons for evaluation, the “Set up your evaluation” button appears. Clicking on this performs two actions. First, it calls netmeta in R to estimate all relative effects from the network and a common heterogeneity parameter. The relative effects are found in the league table, which can be downloaded and saved as a .csv file (“Download league table”). Second, it calls an R function that calculates the contribution matrix (Papakonstantinou). The contribution matrix shows the percentage contribution of information from each study and each direct comparison (shown in columns) to the estimation of each relative effect (shown in rows). It is calculated using the flow decomposition method described in (Papakonstantinou et al., 2018) and is used later in the evaluation of within‐study bias and indirectness. Users can download the output in .csv format using options “Download per study contribution matrix” or “Download per comparison contribution matrix.”

During evaluation, the user can abort computations by pressing the “Cancel” button. Once calculations are done, the “Reset your evaluation” button deletes all previous choices and computations. “Proceed” saves the analysis (CINeMA will remember choices made so far in the case of refreshing or closing and revisiting the page) and takes users to the “Within‐study bias” domain.

### 3.4 Worked example

**Figure 1:**
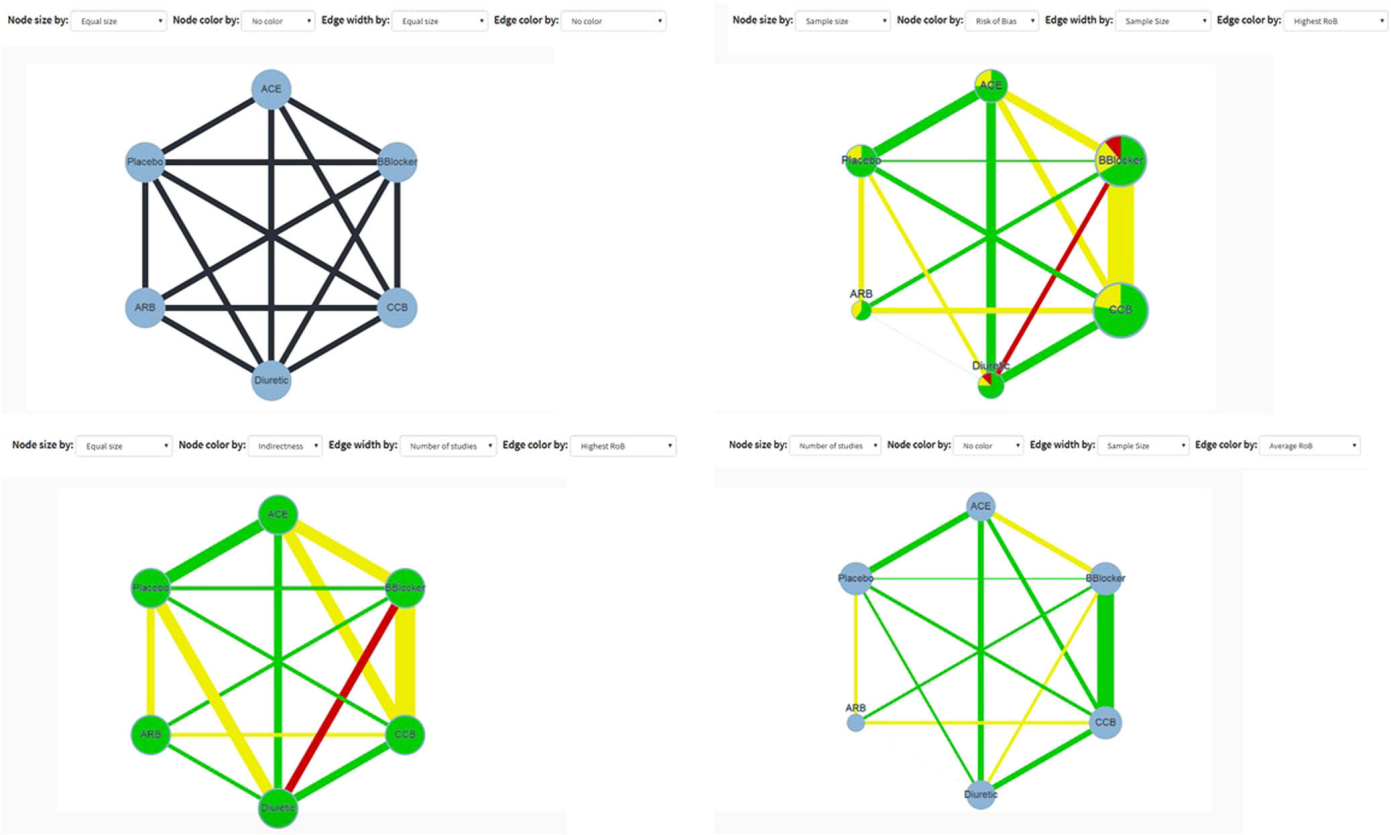
Network plot for the network meta‐analysis of antihypertensive drugs and diabetes incidence using four different sizing and coloring combinations. Green, yellow, and red colors refer to low, moderate, and high risk of bias or indirectness. The plot can be downloaded as a .png file by clicking on “Save Plot” in “Configuration.” ACE, angiotensin‐converting‐enzyme inhibitors; ARB, angiotensin‐receptor blockers; BBlocker, Beta Blocker; CCB, calcium‐channel blocker

The results of selecting different options for weighting the network plot are shown in Figure 1. In the “Define your analysis” section, we select a “Random effects” model and “Odds Ratio” as the effect measure to be analyzed. We select all interventions to be evaluated; note that in this case there is no difference between choosing “Containing any of the above interventions” and choosing “Between the above interventions.” Table 3 shows the downloaded league table; per comparison and per study contribution matrices are given in Appendix Tables A1 and A2.

**Table 3:** NMA results from the network of antihypertensive drugs

ACE	1.070 (0.880, 1.300)	0.713 (0.614, 0.828)	0.846 (0.728, 0.983)	0.665 (0.566, 0.782)	0.885 (0.769, 1.017)
0.935 (0.769, 1.136)	ARB	0.667 (0.557, 0.799)	0.791 (0.664, 0.942)	0.622 (0.504, 0.767)	0.827 (0.691, 0.990)
1.402 (1.208, 1.628)	1.500 (1.252, 1.797)	Beta Blocker	1.186 (1.049, 1.341)	0.933 (0.789, 1.104)	1.240 (1.053, 1.461)
1.182 (1.017, 1.374)	1.264 (1.061, 1.507)	0.843 (0.746, 0.953)	CCB	0.786 (0.670, 0.924)	1.046 (0.894, 1.224)
1.503 (1.279, 1.766)	1.608 (1.303, 1.983)	1.072 (0.906, 1.268)	1.272 (1.083, 1.493)	Diuretic	1.330 (1.124, 1.573)
1.130 (0.983, 1.300)	1.209 (1.011, 1.447)	0.806 (0.684, 0.950)	0.956 (0.817, 1.119)	0.752 (0.636, 0.890)	Placebo

*Note*: Odds ratios and their 95% confidence intervals are presented. Odds ratios <1 favor the intervention specified in the row. The table can be downloaded as a .csv file by clicking on “Download league table” in “Configuration.”

Abbreviations: ACE, angiotensin‐converting‐enzyme inhibitors; ARB, angiotensin‐receptor blockers; CCB, calcium‐channel blocker.

## 4 EVALUATING CONCERNS

### 4.1 Within‐study bias

#### 4.1.1 RoB contributions

The number of studies at low, moderate, and high RoB appears on the top of the page. CINeMA considers the per‐study contribution matrix in conjunction with RoB assessments to evaluate each relative treatment effect with respect to within‐study bias. A bar chart is drawn under the “Risk of bias contributions” section; each bar corresponds to an estimate of relative effect. Each bar also represents a reordering of a column of the per‐study contribution matrix, where studies with low, moderate, and high RoB have been grouped together and colored accordingly. Each study is represented by a colored area with white borders and is proportional to its contribution. Users can download the bar chart as a .png file using the “Save Chart” button.

High RoB should be associated with “Major concerns,” moderate RoB with “Some concerns,” and low RoB with “No concerns.” However, for each relative effect a different combination of high, moderate, and low RoB studies contribute to the estimate. Under the bar graph, a dropdown menu offers three possible “rules” that can be used to summarize the RoB for each relative effect estimate and produce automated judgments. Options include “Majority RoB,” “Average RoB,” and “Highest RoB.” Choosing “Majority RoB” will lead to a level of concern according to the RoB with the greatest total percentage contribution (the greatest block between green, yellow, and red in each bar). The “Highest RoB” will assign a level of concern determined by the highest RoB in each bar. Summarizing RoB assessments using “Average RoB” uses a weighted average score for each relative effect estimate according to the percentage contribution of studies at each bias level. For example, if the contributions from low (arbitrarily assigned a score of 1), moderate (score 2), and high (score 3) RoB studies are 40%, 25%, and 35% respectively, the total RoB score will be 0.40×1+0.25×2+0.35×3=1.95 which rounds to 2 and leads to “Some concerns.” In this example the judgment for “Majority RoB” would be “No concerns” and for “Highest RoB” would be “Major concerns.”

After selecting a rule, the boxes under the dropdown menu—which correspond to the each estimate of relative effects—are colored according to the level of concern, and judgments under each of the three rules are also given in the boxes. Manual change of judgments independently of the applied selection rule is possible; if a judgment is manually changed, the corresponding box is colored gray. “Reset” (the chosen rule) and “Proceed” (to “Reporting bias”) buttons also appear.

#### 4.1.2 Worked example

**Figure 2:**
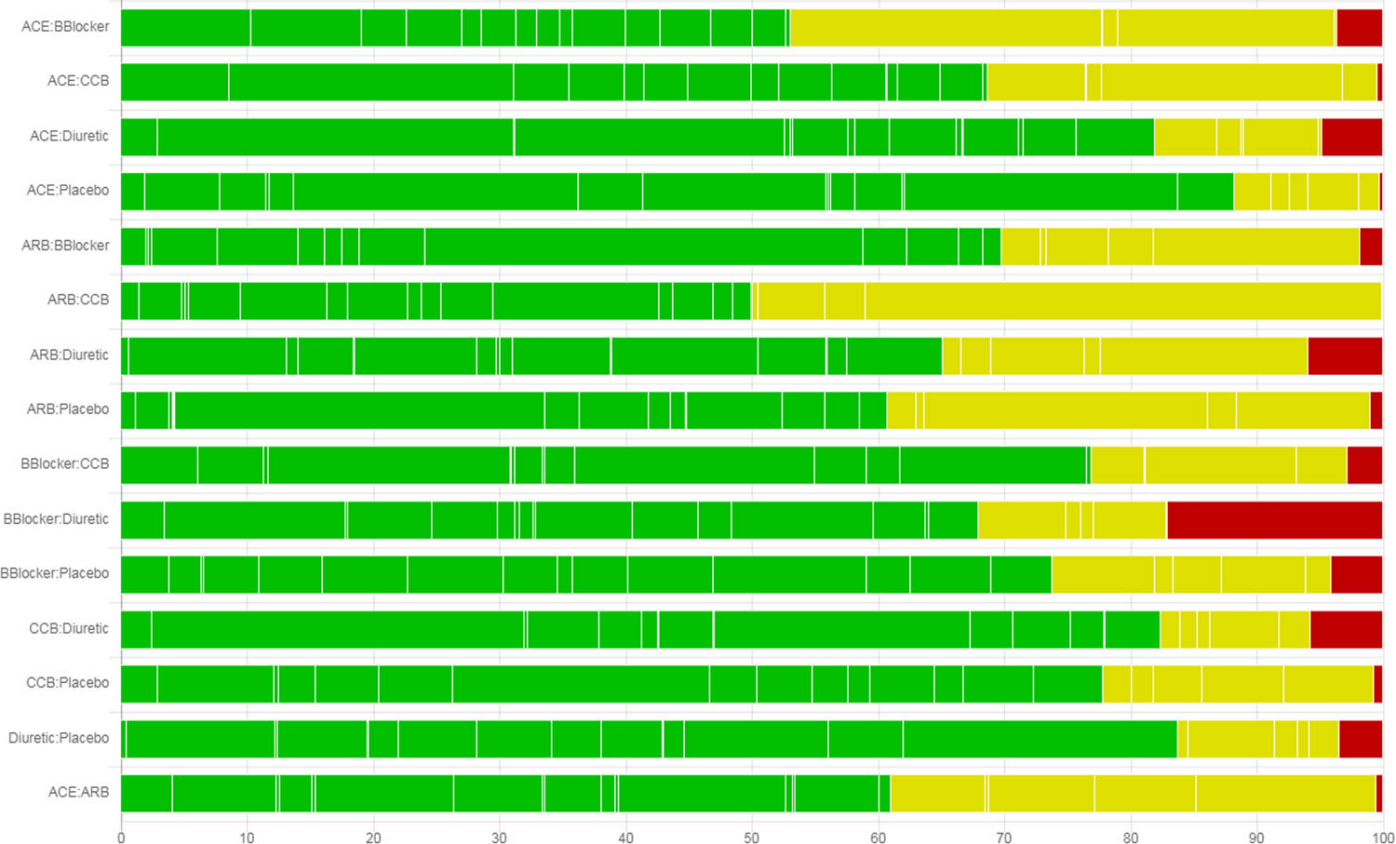
Risk of bias bar chart for the network meta‐analysis of antihypertensive drugs and diabetes incidence. Each bar represents the evidence for a relative treatment effect. White vertical lines separate colored areas which refer to the contribution of each study. Each bar shows the percentage contribution from studies judged to be at low (green), moderate (yellow), and high (red) risk of bias. The plot can be downloaded as a .png file by clicking on “Save Chart” in “Within‐study bias.” ACE, angiotensin‐converting‐enzyme inhibitors; ARB, angiotensin‐receptor blockers; CCB, calcium‐channel blocker

**Figure 3:**
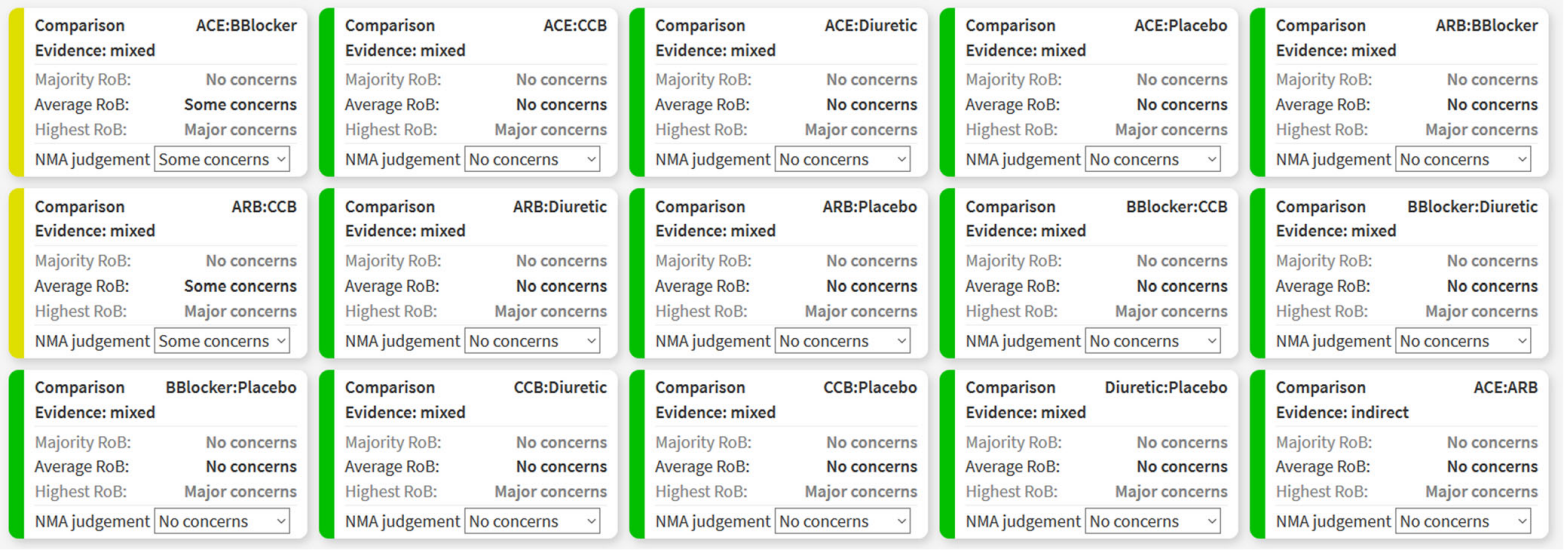
Boxes showing the judgments for within‐study bias for all relative effects in the network meta‐analysis of antihypertensive drugs and diabetes incidence. ACE, angiotensin‐converting‐enzyme inhibitors; ARB, angiotensin‐receptor blockers; CCB, calcium‐channel blocker

Figure 2 shows the bar chart for the worked example. Studies at low RoB contribute 53% in the estimation of ACE versus Beta Blockers, 43% of the contribution comes from studies at moderate RoB, and studies at high RoB contribute the remaining 4%. These RoB contributions resolve into “No concerns,” “Some concerns,” and “Major concerns” using the “Majority RoB,” “Average RoB,” and “Highest RoB” rules respectively. Figure 3 shows the boxes that appear in the software showing the judgments for all relative effects.

### 4.2 Reporting bias

The “Reporting bias” domain refers to biases that can occur due to publication bias, time‐lag bias, selective nonreporting bias, or any other bias that renders the included studies a nonrepresentative sample of the studies undertaken (Dickersin and Chalmers, 2011; Stern and Simes, 1997). Two levels of judgment for reporting bias are suggested: “suspected” and “undetected.” Completeness of the search, considerations related to the particular field (based on existing evidence and empirical studies), and statistical methods undertaken should inform the assessment of reporting bias for each relative treatment effect (Chaimani and Salanti, 2012; Mavridis, Sutton, Cipriani, & Salanti, 2013; Mavridis, Welton, Sutton, & Salanti, 2014).

To facilitate assessment of each effect separately, users can initially “Set all undetected” or “Set all suspected.” They can then change the judgment manually for those estimates that do not fall into the category initially assigned. Note that a manual change from “Suspected” to “Undetected” and vice versa is not considered a deviation from the rule (and relevant boxes are not colored gray) as no rule for reporting bias is implemented. “Reset” and “Proceed” (to the “Indirectness” domain) buttons appear after initial population of judgments. We plan to develop the “Reporting bias” domain of CINeMA further in the months and years to come.

### 4.3 Indirectness

For the indirectness domain, similar to “Within‐study bias,” the summary shows how many studies have been characterized as of low, moderate, and high indirectness at the top of the page. Subsequently, a bar graph shows the contribution of studies at each indirectness level to each NMA estimate. As for the “Within‐study bias” domain, users can select between “Majority,” “Average,” and “Highest” rules to summarize indirectness for each relative effect estimate. Areas are colored accordingly, while judgments under each rule are shown in the boxes. Manual changes can be made, and “Reset” and “Proceed” (to the “Imprecision” domain) buttons appear.

### 4.4 Imprecision

In the CINeMA framework, imprecision is assessed by 95% confidence intervals which may include values that could lead to different clinical conclusions. The calculation of 95% confidence intervals uses the variances of the treatment effects, estimated using maximum likelihood methods, and assumes that treatment effects approximately follow the normal distribution. To judge “Imprecision,” users are asked to define a clinically important size of effect on the scale of the selected effect measure. After pressing “Set,” the range of values to be considered as clinically important appear. The clinically important value and its reciprocal value separate the range of effects into three sets. Between them are effect sizes that favor neither of the compared interventions; on the left and right sides of the two clinically important values one of the two compared interventions is favored. Note that the observed point estimate favors one of the two interventions unless it is exactly on the no‐effect line.

**Figure 4:**
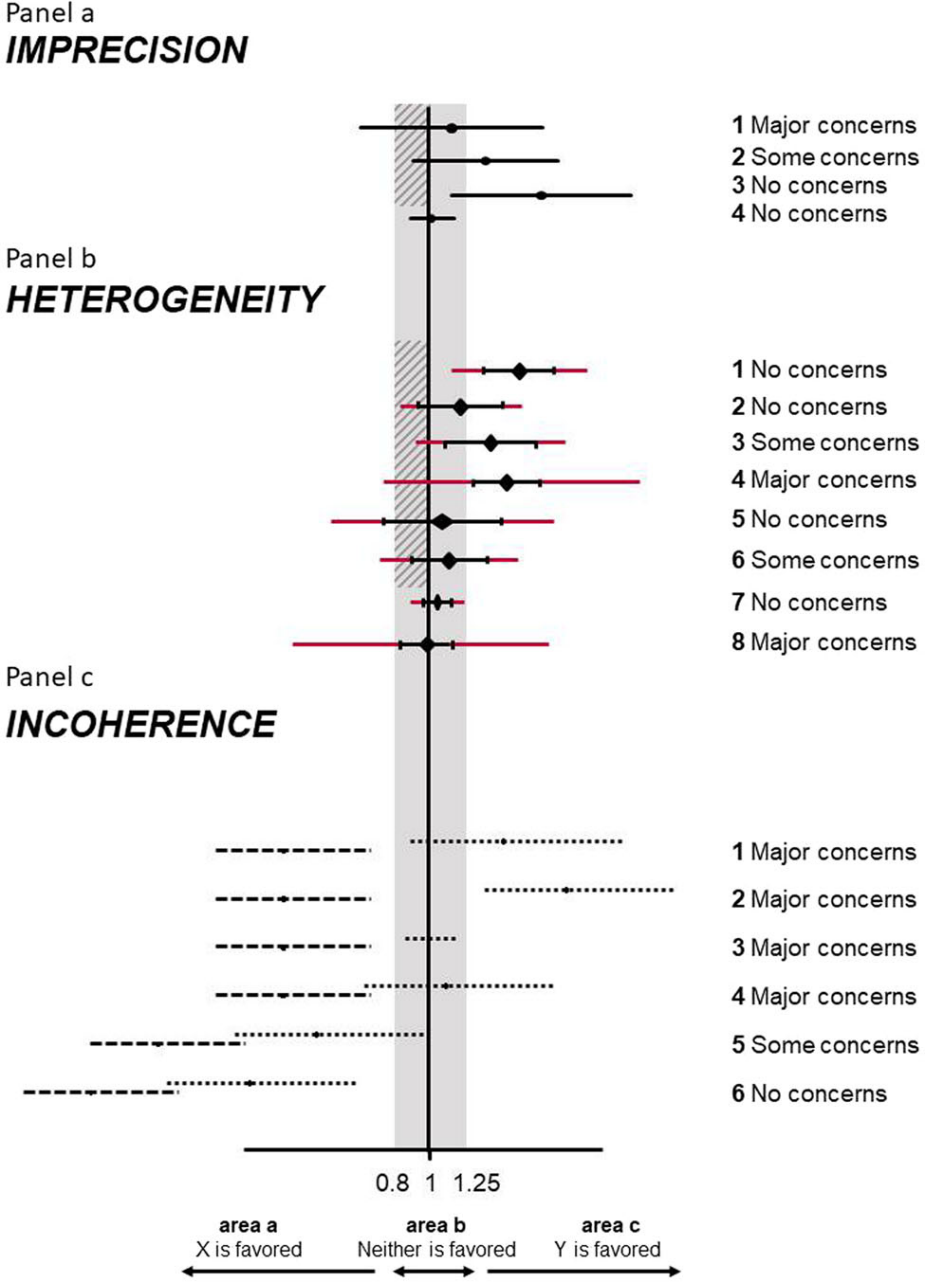
Illustration of rules to assess imprecision (a), heterogeneity (b), and incoherence (c) in CINeMA. We assume several fictional scenarios for the odds ratio from NMA comparing interventions X and Y. The clinically important effects were set at 0.8 and 1.25 ( = 1/0.8). The gray areas represent values that favor neither of the competing interventions. The shaded interval represents the interval between the null effect and clinically important size of effect. Black horizontal lines indicate confidence intervals and red extensions indicate prediction intervals of NMA relative treatment effects. Dotted lines represent direct and dashed lines represent indirect confidence intervals. Judgments are the same for cases symmetrical to those illustrated. NMA, network meta‐analysis

The rules for judging imprecision are based on whether the confidence interval includes the line of no‐effect and the clinically important values. If the confidence interval crosses the line of no‐effect and extends to values that favor the opposite intervention to that favored by the point estimate, “Major concerns” is assigned (Figure 4a). If only the null effect is included in the confidence intervals (and potentially also the clinically important value that favors the same intervention as the point estimate), “Some concerns” is assigned. Finally, “No concerns” is assigned to confidence intervals that only include the clinically important value that favors the same intervention as the point estimate. If the confidence interval lies entirely between the two clinically important values, “No concerns” is assigned (Figure 4a).

After defining the clinically important size of effect, the boxes of the relative treatment effects appear colored according to the default rules described above. Boxes display the 95% (Wald‐type) confidence intervals of the NMA effect, a description of its relation to the clinically important effects, and the judgment, which can be manually altered. Users can also reset the definition of the clinically important size of effect and/or the imprecision judgments with the relevant buttons. The “Proceed” button leads the user to the “Heterogeneity” domain.

#### 4.4.1 Worked example

For illustration, we choose an odds ratio of 1.2 as clinically important and CINeMA informs us that “relative effect estimates below 0.83 and above 1.2 are considered clinically important.” The confidence interval for the comparison between diuretics and placebo ranges from 1.12 to 1.57, which corresponds to case 3 Figure 4a. The automatically generated judgment is “No concerns” and the explanation reads “Confidence interval does not cross clinically important effect.”

### 4.5 Heterogeneity

The importance of heterogeneity depends on the variability of effects (beyond chance) in relation to the clinically important size of effect. The clinically important size of effect is the same as in “Imprecision”; if already specified it will automatically appear on the top of “Heterogeneity.” Otherwise, users need to specify it here; if this is the case, it will also be copied to the “Imprecision” domain. Users can press “Reset” to reset the clinically important effect size; note that this will affect the “Imprecision” domain too.

CINeMA considers the agreement between confidence and prediction intervals to assign a judgment for “Heterogeneity” for each NMA effect. Prediction intervals provide a range within which the true effect of a new study is likely to lie (Riley, Higgins, & Deeks, 2011). It is calculated as $\hat{\mu }+t_{\mathrm{df}}^{a}\sqrt{\tau^{2}+\mathrm{se}{\left(\hat{\mu }\right)}^{2}}$ where $\hat{\mu }$ is the NMA treatment effect, $\mathrm{se}\left(\hat{\mu }\right)$ its standard error and $t_{\mathrm{df}}^{a}$ the $100\left(1-\frac{a}{2}\right)\%$ percentile of the t dustribution with df degrees of freedom. We judge the agreement of conclusions based on confidence and prediction intervals in relation to the null effect and the clinically important effect on the opposite direction to the point estimate Our judgments based on a hypothetical, clinically important odds ratio of 0.8 are summarized and illustrated in Table 4 and in Figure 4a. The automatically produced judgments are shown in boxes. Each box includes the confidence and the prediction interval, a description of their relation to the clinically important effects, and the assigned judgment. The latter can be manually updated.

**Table 4:** Summary of implemented rules for heterogeneity based on the agreement of conclusions between confidence and prediction intervals

Number of crossings of the interval formed by the null effect and the clinically important value that favors the opposite intervention as the point estimate		Prediction intervals		
		0	1	2
Confidence intervals	0	No concerns	Some concerns	Major concerns
	1	NA	No concerns	Some concerns
	2	NA	NA	No concerns

The estimated value of common between‐study variance $\tau^{2}$ is also displayed above the boxes but does not affect automated judgments. It is possible to view “Between‐study variance estimates for each direct comparison along with reference intervals.” To view these, users need to select the types of intervention and outcome and press “View.” Boxes for each relative treatment effect are then updated to include between‐study heterogeneity measures based on direct comparisons ($I^{2}$ and $\tau^{2}$) and reference values for $\tau^{2}$ (first quantile, median, and third quantile). The reference quantiles are taken from empirical studies and are specific to the type of outcome and comparison (Rhodes, Turner, & Higgins, 2015; Turner, Davey, Clarke, Thompson, & Higgins, 2012). Reference quantiles that are lower than the estimated direct $\tau^{2}$ appear in black digits and reference values greater than the estimated $\tau^{2}$ appear in gray digits. The comparison with the reference values does not affect judgments. However, their critical appraisal may lead to changing the automatically generated judgments manually.

#### 4.5.1 Worked example

**Figure 5:**
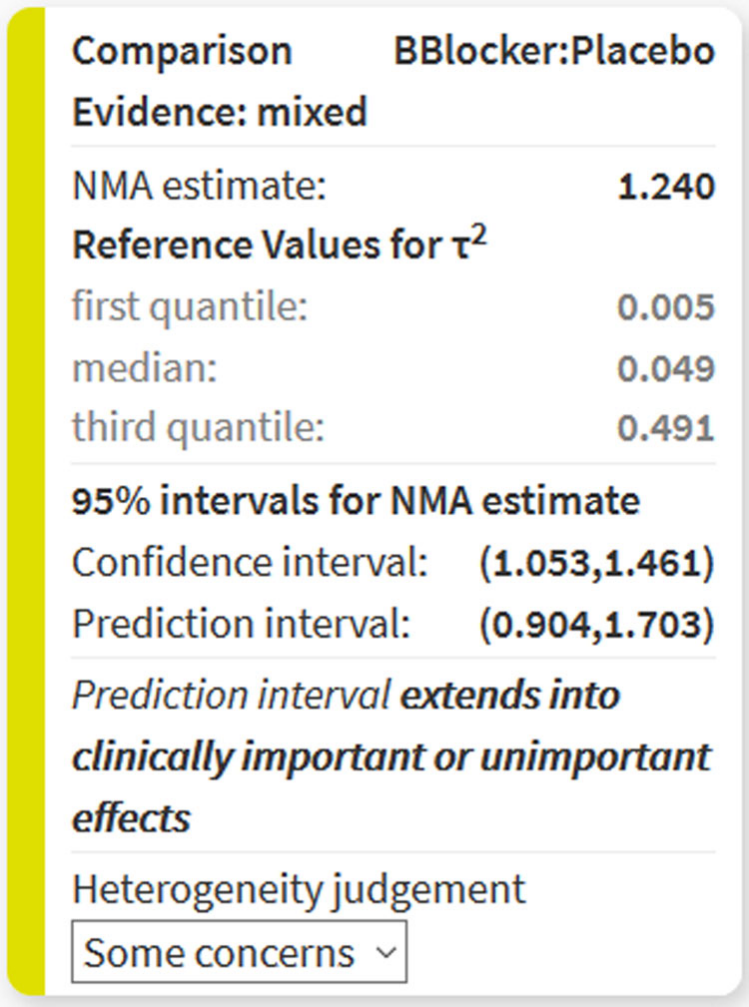
Boxes showing the information for judging heterogeneity for the relative effect of beta blockers versus placebo in the network meta‐analysis of antihypertensive drugs and diabetes incidence

An odds ratio of 1.2 (and 0.83) has already been specified as clinically important. CINeMA reports that “The estimated value of between‐study variance for the network meta‐analysis is 0.016.” The comparison of beta blockers with placebo is judged as case 3 Figure 4a. In particular, “Some concerns” for heterogeneity are assigned, as the confidence interval (1.05–1.46) lies above the interval (0.83–1) while the prediction interval (0.90–1.70) crosses 1. “Prediction interval extends into clinically important or unimportant effects” is given as an explanation in the respective box. Selecting the intervention type (pharmacological for all interventions apart from placebo) and outcome type (semiobjective), boxes are updated to include extra information on heterogeneity (Turner et al., 2012). Figure 5 shows the box that appears in the software referring to the comparison of beta blockers with placebo.

### 4.6 Incoherence

The range of clinically important effects is also considered in the “Incoherence” domain; resetting it using the “Reset” button will affect “Imprecision” and “Heterogeneity” judgments. Incoherence refers to the disagreement between direct and indirect evidence, also called inconsistency in the literature. Several methods have been suggested to assess disagreement statistically (Dias et al., 2013; Dias, Welton, Caldwell, & Ades, 2010; Higgins et al., 2012; White, Barrett, Jackson, & Higgins, 2012). CINeMA performs and displays the results of two methods; the first is a global method to assess incoherence, the design‐by‐treatment interaction test (Higgins et al., 2012; White et al., 2012). Its results ($\chi^{2}$, degrees of freedom, and *p* value) are shown on the top of the “Incoherence” page. In each box, the results of the second method, Separating Indirect from Direct Evidence (SIDE; Dias et al., 2010), are shown, including the relative effect estimate, the direct effect, the indirect effect, the measure of their agreement, and the respective *p* value. As SIDE approach refers to the disagreement measure between direct and indirect evidence as “inconsistency factors,” we also use this terminology. Inconsistency factors measure the disagreement between direct estimates, estimated from studies directly comparing the particular comparison, and indirect estimates, estimated from a NMA including all but the direct studies. Inconsistency factors are given as the ratio of direct and indirect effects if a ratio measure is used, or as a difference otherwise.

The rules used to produce automatic judgments are as follow:

(1) Effect estimates based on both direct and indirect evidence and with a *p* value from SIDE greater than 0.10 are assigned “No concerns.”
(2) To assign judgments for effect estimates with both direct and indirect evidence and with a *p* value from SIDE <0.10, areas a, b, and c are defined as illustrated in Figure 4a (below, within, and above the clinically important effects). The confidence intervals for the direct and indirect evidence are then compared with these areas and incoherence judged according to Table 5. Figure 4c illustrates six examples of such judgments.
  **Table 5:** Summary of implemented rules for incoherence based on the agreement of direct and indirect estimates with their 95% confidence intervals in the areas defined in Figure 4c

Common areas	Incoherence judgment
0	Major concerns
1	Major concerns
2	Some concerns
3	No concerns
(3) Effect estimates based only on direct evidence or indirect evidence are assigned a judgment determined by the *p* value of the design‐by‐treatment interaction test: if the *p* value is <0.05 then “Major concerns,” if between 0.05 and 0.10 then “Some concerns,” otherwise “No concerns.” If the design‐by‐treatment interaction test is not estimable (because the network does not have any closed loop of evidence) then “Major concerns” are assigned to all comparisons.

As with other domains, judgments can be updated manually and a “Reset” and “Proceed” (to “Report”) button appear if clinically important size of effect is set.

#### 4.6.1 Worked example

**Figure 6:**
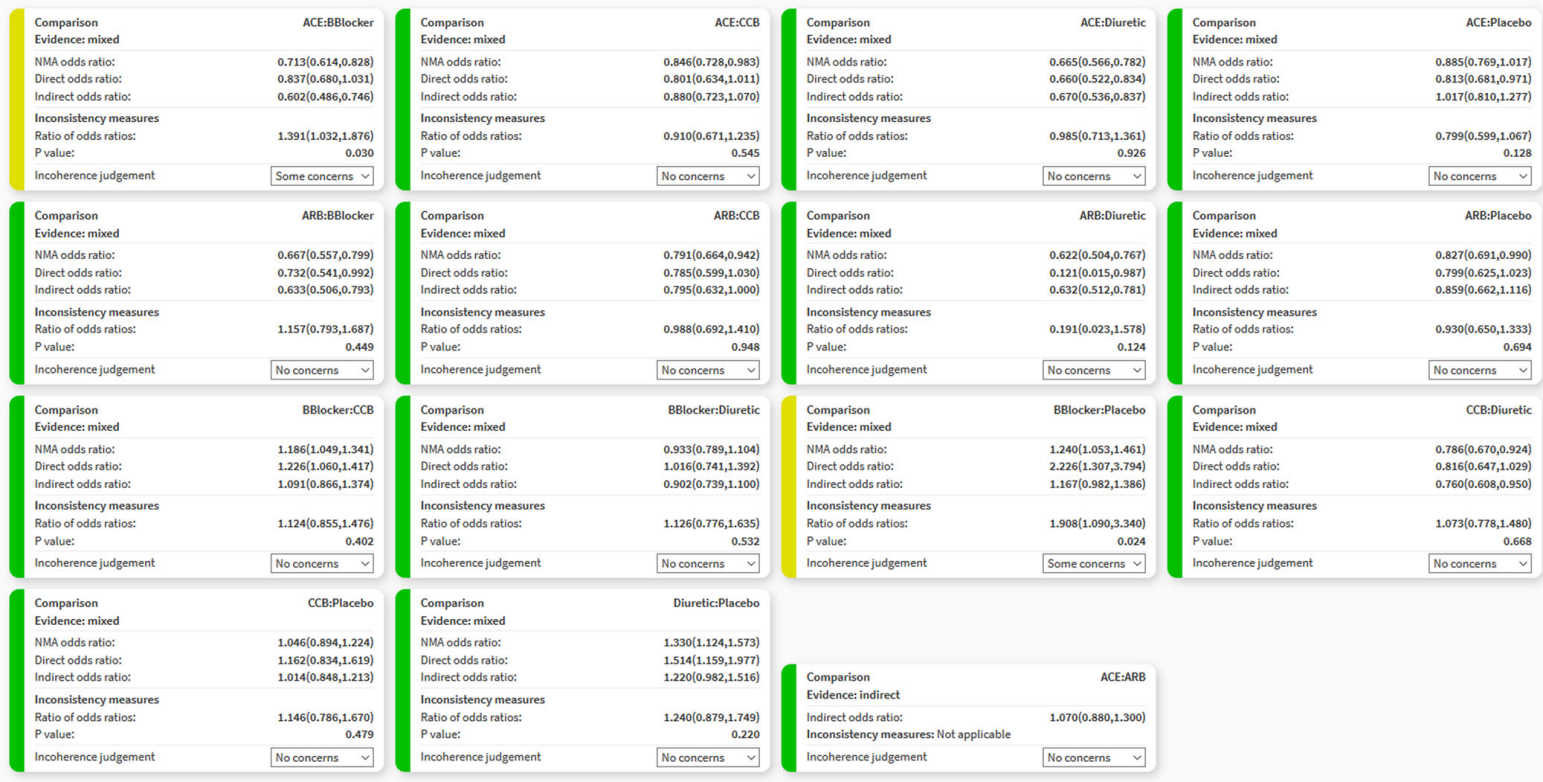
Boxes showing the judgments for incoherence for all relative effects in the network meta‐analysis of antihypertensive drugs and diabetes incidence. ACE, angiotensin‐converting‐enzyme inhibitors; ARB, angiotensin‐receptor blockers; CCB, calcium‐channel blocker

As in case 5 of Figure 4c, “Some concerns” apply to the ACE inhibitor versus Beta Blockers comparison with respect to “Incoherence.” Confidence intervals of both direct (0.68–1.03) and indirect (0.49–0.75) treatment effects extend below the clinically important effects zone, only the direct effect's confidence interval lies within the (0.83–1.2) interval and none extend above 1.2; thus, direct and indirect treatment effects do not have substantial, but only minor disagreement (Table 5). Figure 6 shows the boxes that appear in the software showing the judgments for all relative effects for “Incoherence.”

## 5 DISPLAYING JUDGMENTS FOR ALL SIX DOMAINS: *REPORT*

The “Report” page brings together all the judgments for the six domains across all evaluated treatment effects. Relative effects informed by only direct or both direct and indirect evidence are shown first, followed by relative effects informed only by indirect evidence. A thick gray left border line appears for judgments whose automatically generated judgments have been manually modified. Users can visit the “Report” page as soon as at least one domain has been assessed. If users wish to summarize judgments across domains, the “Confidence rating” dropdown menu can be used to manually assign an overall level of confidence to each relative effect. The default judgment is “High” confidence; downgrading by one, two, or three levels will lead to a confidence rating of “Moderate,” “Low,” or “Very low” respectively. We recommend considering judgments on different domains jointly rather than in isolation (Nikolakopoulou et al., 2019; Salanti et al., 2014). For example, “Indirectness” and “Incoherence” domains are closely related, as they both refer to considerations of similarity across included studies which could or could not manifest statistically in the data. “Imprecision” and “Heterogeneity” are also related as big heterogeneity will also affect the precision of relative treatment effects. By clicking “Reset” all judgments are set to “High.” Users can also download the final report as a .csv file by clicking on “Download report.”

### 5.1 Worked example

**Figure 7:**
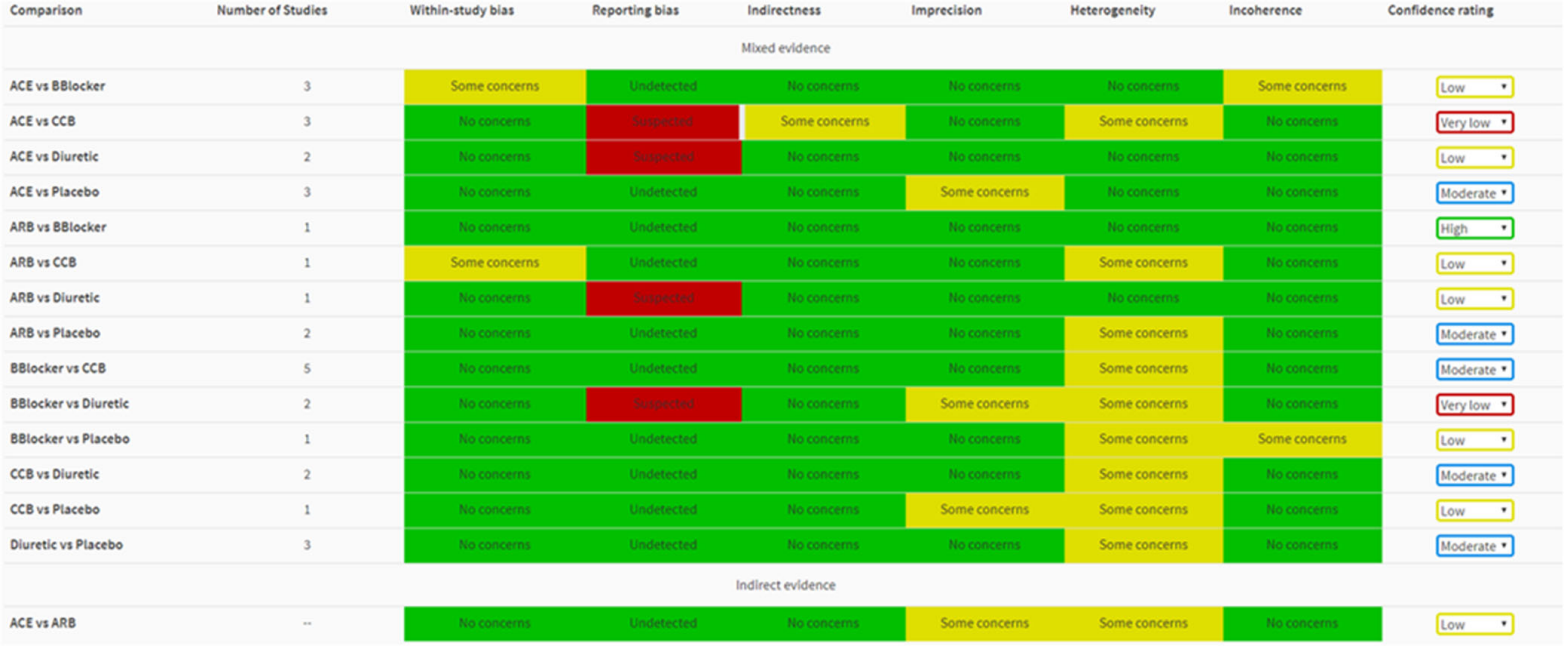
Final output of CINeMA for the network of antihypertensive drugs and incidence of diabetes. The table shows the level of concern for each of the six domains for each comparison and can be downloaded as a .csv file by clicking on “Download Report” in “Report.” ACE, angiotensin‐converting‐enzyme inhibitors; ARB, angiotensin‐receptor blockers; CCB, calcium‐channel blocker

The report of the judgments for the Elliot et al. network is shown in Figure 7. Note that some of the choices are made for the sake of illustration only; for example, indirectness data are fictional, reporting bias judgments are illustrative, and the clinically important odds ratio of 1.2 is not justified by clinical or epidemiological reasoning. A thick gray left border appears for the judgment of indirectness for the comparison of ACE versus CCB inhibitors as the judgment was manually changed.

## 6 DISCUSSION

We have outlined how CINeMA software can be used to facilitate evaluation of results of NMA. Such an evaluation is an important but challenging part of a systematic review with multiple interventions. CINeMA, with semiautomation of methods via a guided on‐line process greatly simplifies this process, particularly for large networks. CINeMA is freely available and open‐source and no login is required. It is largely based on the methodological framework described previously (Nikolakopoulou et al., 2019; Salanti et al., 2014). While the main guiding principles of the CINeMA framework have been established (Nikolakopoulou et al., 2019; Salanti et al., 2014), specific methods, recommendations, and implementation of automated rules in CINeMA software are evolving.

Important platform updates will follow. These include users being able to upload multiple projects they are working on concurrently. With the addition of this feature, users will be able to also have multiple outcomes per project, something that is currently not supported by CINeMA. Note, however, that dependency between outcomes will not be assessed. Users will also be able to download a league table with studies of only low, or only low and moderate RoB (or indirectness). The “Report” page will be updated so that users can click on each comparison by domain judgment and decide whether they will downgrade their confidence or not, and if yes, for one or two levels.

Subjectivity is inevitable in any process or system evaluating evidence, and CINeMA is no exception. Several aspects of the evaluations such as interpretation of heterogeneity and definition of a clinically important size of effect are clearly subjective. A study to examine CINeMA's reproducibility by measuring the agreement between assessments made by researchers using CINeMA would be of interest. The clearly defined criteria on which judgments are based should increase reproducibility and the fact that CINeMA is open‐source ensures transparency.

Evidence synthesis is used by organizations to take decisions about whether to reimburse a medicinal product, by clinical guideline panels to recommend one drug over another, and by clinicians to prescribe an intervention or recommend a diagnostic procedure. CINeMA is a transparent framework to evaluate evidence from systematic reviews with multiple interventions, and we hope that the software presented here will facilitate its uptake.

## APPENDIX A

**Table A1:** Per‐comparison contribution matrix for the network of antihypertensive treatments

NMA relative treatment effect/comparisons	ACE: BBlocker	ACE: CCB	ACE: Diuretic	ACE: Placebo	ARB: BBlocker	ARB: CCB	ARB: Diuretic	ARB: Placebo	BBlocker: CCB	BBlocker: Diuretic	BBlocker: Placebo	CCB: Diuretic	CCB: Placebo	Diuretic: Placebo
Mixed estimates														
ACE: BBlocker	43.35	10.79	6.735	7.2182	2.6733	0.1015	0.014	2.7608	14.5348	5.345	2.19	2.01	1.6333	0.634
ACE: CCB	13.545	33.55	8.34	9.0103	0.062	2.767	0.015	2.69	14.6583	0.632	0.5433	8.41	5.06	0.717
ACE: Diuretic	8.6417	8.425	40.46	11.2922	0.028	0.283	0.1067	0.3617	1.5792	7.085	0.0505	10.7972	0.51	10.37
ACE: Placebo	5.2763	6.0767	6.81	58.63	1.85	1.65	0.018	3.518	0.918	0.3833	2.125	0.2147	5.13	7.39
ARB: BBlocker	5.5792	0.1325	0.05	5.3967	34.71	16.42	0.275	11.28	17.6033	2.6025	2.19	0.0575	1.3733	2.32
ARB: CCB	0.1495	3.81	0.3925	4.352	13.19	40.87	0.28	12.1037	13.7708	0.112	0.5433	3.2078	4.785	2.4233
ARB: Diuretic	2.5133	1.4997	8.1997	4.1867	11.5933	16.4067	0.94	17.0497	0.468	8.66	0.048	15.62	0.245	12.57
ARB: Placebo	3.91	3.0067	0.327	7.2437	7.6403	10.6067	0.27	51.75	0.172	1.4333	2.125	2.367	5.405	3.7433
BBlocker: CCB	7.4083	5.98	0.6067	0.8217	4.0908	4.015	0.0033	0.0725	64.55	4.05	1.38	4.7375	2.1967	0.0775
BBlocker: Diuretic	12.25	1.2375	12.62	0.8675	2.6883	0.15	0.195	2.3433	17.6942	24.86	2.07	15.48	1.1267	6.4075
BBlocker: Placebo	14.35	2.83	0.3075	17.4875	6.785	2.09	0.0025	8.8775	14.8417	5.92	8.64	2.3367	7.585	7.9467
CCB: Diuretic	2.6428	8.35	10.7133	0.2795	0.032	2.4653	0.19	2.2433	11.4542	8.38	0.4633	41.2	4.295	7.2812
CCB: Placebo	4.15	10.225	0.7625	15.1375	1.7433	7.15	0.005	8.8983	9.8208	0.9875	2.94	8.785	20.38	9.005
Diuretic: Placebo	1.4	1.25	13.42	16.07	1.7108	2.3433	0.2	4.2542	0.2675	5.0883	2.245	9.7558	5.895	36.1
Indirect estimates														
ACE: ARB	13.19	11.055	4.855	18.2	13.2933	14.21	0.31	19.4867	0.71	0.8133	0	2.31	0.135	1.4217

*Note*: Columns refer to comparisons with direct data and rows refer to NMA relative treatment effects. Entries show how much each comparison contributes to the estimation of NMA relative treatment effects. The table can be downloaded as a .csv file by clicking on “Download per comparisons contribution matrix” in “Configuration.”

Abbreviations: ACE, angiotensin‐converting‐enzyme inhibitors; ARB, angiotensin‐receptor blockers; BBlocker, Beta Blocker; CCB, calcium‐channel blocker.

**Table A2:** Per‐study contribution matrix for the network of antihypertensive treatments

NMA treatment effect/study IDs	1	2	3	4	5	6	7	8	9	10	11
Mixed estimates											
ACE: BBlocker	10.3307	8.8137	0.014	3.5622	4.3208	24.5731	1.5633	2.7752	0.1198	1.633	3.6652
ACE: CCB	8.5763	22.566	0.015	4.4111	4.3575	7.678	1.5232	3.4642	0.1354	5.06	0.4334
ACE: Diuretic	2.966	28.1532	0.1067	21.3995	0.4694	4.8986	0.2048	4.3415	1.9589	0.51	4.8583
ACE: Placebo	1.9547	5.9190	0.018	3.6018	0.2729	2.9909	1.992	22.5414	1.396	5.13	0.2629
ARB: BBlocker	2.0761	0.1095	0.275	0.0264	5.233	3.1626	6.3872	2.0749	0.4382	1.373	1.7846
ARB: CCB	1.4515	3.4458	0.28	0.2076	4.0937	0.0847	6.8536	1.6732	0.4578	4.785	0.0768
ARB: Diuretic	0.7138	12.4406	0.94	4.3368	0.1391	1.4247	9.6542	1.6096	2.3744	0.245	5.9383
ARB: Placebo	1.1829	2.6439	0.27	0.173	0.0511	2.2164	29.3029	2.785	0.7071	5.405	0.9829
BBlocker: CCB	6.1387	5.253	0.0033	0.3209	19.189	4.1994	0.0411	0.3159	0.0146	2.197	2.7772
BBlocker: Diuretic	3.4497	14.3396	0.195	6.6748	5.26	6.944	1.3269	0.3335	1.2104	1.127	17.0469
BBlocker: Placebo	3.9049	2.5437	0.0025	0.1626	4.412	8.1344	5.0268	6.7234	1.5011	7.585	4.0594
CCB: Diuretic	2.4724	29.5536	0.19	5.6663	3.405	1.4981	1.2703	0.1075	1.3754	4.295	5.7463
CCB: Placebo	2.9389	9.2002	0.005	0.4033	2.9195	2.3524	5.0386	5.8199	1.701	20.38	0.6771
Diuretic: Placebo	0.4631	11.8137	0.2	7.0979	0.0795	0.7936	2.4089	6.1784	6.8192	5.895	3.4892
Indirect estimates											
ACE: ARB	4.1417	8.194	0.31	2.5678	0.2111	7.4768	11.0341	6.9973	0.2685	0.135	0.5577

NMA treatment effect/study IDs	12	13	14	15	16	17	18	19	20	21	22
Mixed estimates											
ACE: BBlocker	1.7862	0.9888	4.2769	2.673	4.0015	3.3405	2.6568	1.1975	0.3826	17.213	0.102
ACE: CCB	2.2297	4.1373	4.3133	0.062	0.8908	3.3688	3.3164	1.1668	0.4327	19.095	2.767
ACE: Diuretic	2.7944	5.3117	0.4647	0.028	4.4307	0.3629	4.1563	0.1569	6.2576	5.8771	0.283
ACE: Placebo	14.5088	0.1056	0.2701	1.85	3.7802	0.211	21.5798	1.526	4.4594	3.9696	1.65
ARB: BBlocker	1.3355	0.0283	5.1799	34.71	3.4897	4.0457	1.9863	4.8928	1.4	3.5611	16.42
ARB: CCB	1.077	1.5781	4.0521	13.19	1.0817	3.1649	1.6018	5.2501	1.4623	3.2522	40.87
ARB: Diuretic	1.036	7.6843	0.1377	11.59	5.3801	0.1076	1.541	7.3955	7.5852	1.316	16.41
ARB: Placebo	1.7925	1.1644	0.0506	7.640	3.3529	0.0395	2.6662	22.4471	2.2589	2.2608	10.61
BBlocker: CCB	0.2033	2.3306	18.9941	4.091	2.6689	14.8351	0.3024	0.0314	0.0468	12.0214	4.015
BBlocker: Diuretic	0.2147	7.6154	5.2066	2.688	11.2137	4.0665	0.3193	1.0164	3.8665	5.7249	0.15
BBlocker: Placebo	4.3275	1.1495	4.3672	6.785	12.1509	3.411	6.4366	3.8507	4.7953	6.5805	2.09
CCB: Diuretic	0.0692	20.2684	3.3704	0.032	4.6091	2.6324	0.1029	0.9731	4.3937	5.4936	2.465
CCB: Placebo	3.746	4.3218	2.8898	1.743	5.1204	2.2571	5.5716	3.8597	5.4339	6.4603	7.15
Diuretic: Placebo	3.9767	4.7994	0.0787	1.711	11.3410	0.0615	5.9148	1.8453	21.784	0.9058	2.343
Indirect estimates											
ACE: ARB	4.5038	1.1364	0.2089	13.29	0.5508	0.1632	6.6988	8.4525	0.8579	8.0201	14.21

*Note*: Columns refer to studies and rows refer to NMA relative treatment effects. Entries show how much each study contributes to the estimation of NMA relative treatment effects. The table can be downloaded as a .csv file by clicking on “Download per study contribution matrix” in “Configuration.”

Abbreviations: ACE, angiotensin‐converting‐enzyme inhibitors; ARB, angiotensin‐receptor blockers; BBlocker, Beta Blocker; CCB, calcium‐channel blocker.
